# In the Sick Room

**Published:** 1890-03

**Authors:** 


### IN THE SICK ROOM.

Nothing is gained, and much time that is very valuable is wasted, by allowing ourselves to become nervous and unable to be of the slightest use in the sick room.

Although we may consider a person too ill to be aware of what is taking place about him, he is oftentimes fully cognizant of the merest trifles, and always more or less susceptible to any and all things going on. For that reason, conversation about the condition of the patient carried on in whispers, or in any mysterious manner, should be avoided and an air of quiet cheerfulness always maintained.

Nothing is so annoying as to be continually asked if we do not wish the pillows changed, the bed clothes straightened, the blinds closed or opened, some nourishment brought, or any small details attended too. Better, by far, to see for one's self, and do quietly without disturbing the patient. Particularly if they are disposed to sleep, do not at once get a newspaper of the kind that has the greatest possible amount of rattle in it, and seat thyself in a rocking chair regardless of the possible effect it may have upon the nervous condition of thy friend.

When it is time for nourishment, or medicine, be prompt to give it, but always without talking it over too much; and if it is the bitter cup that is to be prescribed, have something agreeable to follow, and a cheery word. If it is the food or broth, have it prepared outside the sick room, and brought quietly and above all, in an attractive form, bearing in mind that a little, daintily presented, will be much more acceptable, and partaken of with more benefit than a larger quantity.

An invalid is oftentimes better nourished by partaking of a little sustenance at short intervals, and the manner in which one is cared for has much to do with their improvement.—*Good Housekeeping.*

